# Glycemic Status and Effect of Immediate Intensive Statin on Mild Ischemic Stroke: A Subgroup Analysis of the INSPIRES Trial

**DOI:** 10.1002/cns.70882

**Published:** 2026-04-27

**Authors:** Lingling Jiang, Zihao Deng, Ying Gao, Xuan Wang, Yingying Yang, Tingting Wang, Yuesong Pan, Weiqi Chen, Yilong Wang

**Affiliations:** ^1^ China National Clinical Research Center for Neurological Diseases, Beijing Tiantan Hospital Capital Medical University Beijing China; ^2^ Department of Neurology, Beijing Tiantan Hospital Capital Medical University Beijing China; ^3^ National Center for Neurological Disorders Beijing China; ^4^ Advanced Innovation Center for Human Brain Protection Capital Medical University Beijing China; ^5^ Chinese Institute for Brain Research Beijing China; ^6^ Beijing Laboratory of Oral Health Capital Medical University Beijing China; ^7^ Beijing Municipal Key Laboratory of Clinical Epidemiology Capital Medical University Beijing China

**Keywords:** atherosclerosis, diabetes mellitus, intensive statin, ischemic stroke

## Abstract

**Aims:**

A subgroup analysis of the Intensive Statin and Antiplatelet Therapy for High‐risk Intracranial or Extracranial Atherosclerosis (INSPIRES) trial to evaluate intensive statin effects in patients with ischemic stroke by glycemic status.

**Method:**

Patients were stratified into three subgroups based on glycemic status: without type 2 diabetes mellitus (T2DM), with newly diagnosed T2DM, with a history of T2DM. The primary outcomes included stroke and moderate‐to‐severe bleeding, whereas secondary outcomes comprised composite vascular events, poor functional outcomes, and any bleeding within 90 days.

**Results:**

Among 6100 patients, 4038 were without T2DM, 404 had a newly diagnosed T2DM, and 1658 had a history of T2DM. No significant risk reduction of stroke was observed with immediate versus delayed statin therapies across glycemic subgroups (*p* for interaction = 0.29). Compared to delayed‐intensive statin, immediate‐intensive statin treatment was associated with reduced risk of poor functional outcome in non‐T2DM patients (adjusted relative risk 0.71, 95% CI 0.57–0.90; *p* = 0.004), but no benefit in those with newly diagnosed T2DM or those with a history of T2DM (*p* for interaction = 0.003). There was no glycemic status by treatment interaction for risk of bleeding or composite vascular event.

**Conclusions:**

Patients without T2DM derived greater benefit in functional outcomes from immediate‐intensive statin after ischemic stroke than those with T2DM.

## Graphical abstract

Immediate‐intensive statin improves functional outcomes in non‐diabetic stroke patients: glycemic status modulates intensive statin treatment response. Early intensive statin therapy should be prioritized in stroke patients without diabetes, whereas alternative strategies may be needed for long‐standing T2DM to optimize functional recovery.

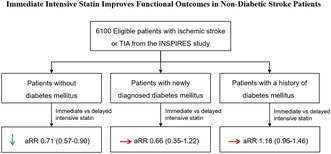

## 1 Introduction

Ischemic stroke remains a leading cause of global disease burden [1, 2], contributing to adverse effects including early neurological decline, recurrent strokes, and long‐term disability [3, 4]. Given its high occurrence, optimizing secondary prevention strategies is of critical importance. The use of statins, notably in high‐intensity regimens, is essential for secondary prevention by effectively lowering low‐density lipoprotein cholesterol (LDL‐C) levels, stabilizing atherosclerotic plaques, and improving endothelial function [5, 6, 7, 8, 9]. Evidence from the Stroke Prevention by Aggressive Reduction in Cholesterol Levels (SPARCL) study demonstrated that high‐intensity statin treatment yields a significant reduction in risk of recurrent stroke, supporting its routine use for secondary stroke prevention [10]. However, individual response to statin therapy varies among patients, highlighting the necessity for personalized approaches, particularly in patients with dysglycemia [11, 12, 13].

Diabetes mellitus (DM) is a well‐established risk factor for unfavorable outcomes in ischemic stroke, with increased risks of recurrent events and mortality [14, 15, 16]. Emerging evidence suggests that the efficacy of lipid‐lowering therapy may differ according to glycemic status [17, 18]. A secondary analysis of the SPARCL trial indicated that patients with type 2 diabetes mellitus (T2DM) faced higher risks for recurrent stroke and cardiovascular events; the relative benefit of statin therapy did not differ between those with or without T2DM [19]. Studies demonstrated that vascular damage began before insulin resistance and clinical diagnosis, ultimately progressing to severe micro‐ and macrovascular complications [20]. The long diabetes duration exhibited an obvious association with elevated stroke risk, independent of conventional cardiovascular risk factors [21, 22]. Moreover, patients with established T2DM exhibit higher 90‐day stroke risk compared with non‐diabetic individuals, whereas those with newly diagnosed T2DM do not show the same magnitude of risk elevation [23]. These observations suggested that the impact of diabetes on vascular risk and therapeutic response may vary by disease stage. According to the 2020 European Heart Journal guidelines, it is recommended to balance the benefits of statins against potential metabolic risks in the diabetic population [24].

The Intensive Statin and Antiplatelet Therapy for Acute High‐Risk Intracranial or Extracranial Atherosclerosis (INSPIRES) trial recently provided evidence that initiating intensive statin therapy within 72 h of stroke onset, rather than 3‐day delayed‐intensive statin therapy, reduced the risk of poor functional outcome in mild ischemic stroke patients [25]. Nevertheless, it remains unclear whether these benefits extend uniformly across different glycemic subgroups. Considering the complex interactions between dysglycemia, statin metabolism, and cerebrovascular dynamics, it is crucial to address this question to advance precision stroke medicine.

In this subgroup analysis of the INSPIRES trial, we aimed to investigate whether the efficacy of immediate‐intensive statin therapy in patients with mild ischemic stroke varied according to glycemic status (categorized as without diabetes mellitus, with newly diagnosed diabetes mellitus, and with a history of diabetes mellitus). We hypothesized that the response to statin treatment would differ across glycemic subgroups.

## 2 Methods

### 2.1 Study Design and Participants

This was a subgroup analysis of the INSPIRES trial, a rigorously designed study conducted in accordance with the Consolidated Standards of Reporting Trials (CONSORT) guidelines. Briefly, the INSPIRES trial was a nationwide multi‐center, double‐blind, randomized, placebo‐controlled 2 × 2 factorial trial. The trial was carried out across 222 clinical sites in China between September 17, 2018, and October 15, 2022. The trial design rationale and detailed methods have been previously described [26]. The trial enrolled 6100 patients with mild acute ischemic stroke or high‐risk transient ischemic attack (TIA) within 72 h of symptom onset, to evaluate the efficacy and safety of two therapeutic strategies: (1) dual antiplatelet therapy (clopidogrel plus aspirin) versus aspirin alone; (2) immediate‐intensive statin vs. 3‐day delayed‐intensive statin regimen. Ethical approval was obtained from the ethics committees at Beijing Tiantan Hospital (KY2017‐065‐02) and from each participating center. Written informed consent was provided by all participants or their legal representatives prior to enrollment. The trial was registered at ClinicalTrials.gov (NCT03635749).

In the INSPIRES trial, eligible participants were adults aged 35–80 years who met one of the following clinical criteria: (1) mild acute ischemic stroke, National Institutes of Health Stroke Scale (NIHSS) (higher scores indicate greater stroke severity) score ≤ 5, occurring 24–72 h after symptom onset; (2) high‐risk TIA, ABCD^2^ score ≥ 4, also within 24–72 h of symptom onset; or (3) ischemic stroke with an NIHSS score of 4 or 5, presenting within 24 h of symptom onset. Additionally, participants were required to satisfy at least one imaging criterion confirming atherosclerotic origin: (1) 50% or higher stenosis of a major intracranial or extracranial artery, identified by carotid ultrasound or vascular imaging and considered causally related to the clinical symptoms and infarction; or (2) acute multiple infarcts attributed to large‐artery atherosclerosis, including cases with ipsilateral non‐stenotic unstable plaques, as documented by cranial computed tomography (CT) or magnetic resonance imaging (MRI). Patients were excluded if they had received intravenous thrombolytic therapy, mechanical thrombectomy, defibrillation, or used anticoagulation and antiplatelet agents other than clopidogrel or aspirin. Additional exclusion criteria comprised a confirmed diagnosis of cardiogenic ischemic cerebrovascular disease, non‐vascular intracranial pathology, history of intracranial hemorrhage, use of dual antiplatelet therapy (DAPT) within 14 days prior to randomization, or severe liver or renal dysfunction at baseline. A complete list of inclusion and exclusion criteria is available in the study protocol [26].

### 2.2 Procedures

Eligible participants were randomly allocated in a 1:1:1:1 ratio to one of four treatment groups within 72 h of stroke onset: (1) intensive antiplatelet with immediate intensive statin; (2) intensive antiplatelet with delayed intensive statin; (3) standard antiplatelet with immediate intensive statin; and (4) standard antiplatelet with delayed intensive statin. In the main trial, no significant interaction was observed between antiplatelet and statin treatments on primary efficacy outcome (*p* for interaction = 0.16 for stroke), supporting the validity of separately analyzing the effects of statin therapy. The present subgroup analysis focused specifically on the effect of statin treatment across different glycemic status groups.

The statin regimens were as follows. In the immediate‐intensive statin group, participants received atorvastatin on 80 mg/day on Days 1–21, then 40 mg/day on Days 22–90. In the delayed‐intensive statin group, atorvastatin placebo was administered on Days 1–3; from 4 to 21 days, participants received atorvastatin placebo with atorvastatin 40 mg/day; and from 22 to 90 days, atorvastatin 40 mg/day alone was given. After the trial period, management was determined by local investigators based on the most current clinical guidelines.

Baseline clinical data including demographic information, medical history, concomitant medication use, and laboratory examination of participants were collected at the time of enrollment by trained research coordinators. Patients were stratified into three subgroups according to glycemic status: without T2DM, with newly diagnosed T2DM, and with a history of T2DM. A history of T2DM was determined by either a self‐reported physician diagnosis prior to enrollment or current use of glucose‐lowering agents. Newly diagnosed T2DM was identified during hospitalization in patients without prior glycemic abnormalities, based on fasting glucose ≥ 7.0 mmol/L, 2‐h post‐load glucose ≥ 11.1 mmol/L, or HbA1c ≥ 6.5%, with careful exclusion of stress hyperglycemia.

### 2.3 Clinical Outcomes

The primary efficacy outcome was the occurrence of any new ischemic or hemorrhagic stroke within 90 days (within a window of ±7 days). The secondary efficacy outcomes consisted of composite cardiovascular events (stroke, myocardial infarction, or cardiovascular death) and ischemic stroke within 90 days, along with poor functional outcome at 90 days, defined as a modified Rankin Scale (mRS) score of 2–6. For safety, the primary outcome was moderate‐to‐severe bleeding within 90 days, defined by criteria from the Global Utilization of Streptokinase and Tissue Plasminogen Activator for Occluded Coronary Arteries trial [27]. Secondary safety outcomes included hepatotoxicity (alanine aminotransferase [ALT] or aspartate aminotransferase [AST] levels exceeding 3 times the upper limit of normal), any bleeding, and all‐cause death within 90 days. All endpoint analyses utilized events adjudicated by an independent clinical event committee.

### 2.4 Statistical Analysis

This subgroup analysis included all participants from the INSPIRES study. Baseline characteristics including demographic data, medical history, and relevant risk factors were summarized using descriptive statistics as appropriate, stratified by glycemic status (without T2DM, with newly diagnosed T2DM, and with a history of T2DM) and statin therapy (immediate‐intensive statin vs. delayed‐intensive statin). Continuous variables were reported as median with interquartile range (IQR), whereas categorical variables were presented as frequencies and percentages. Group comparisons were performed using the Kruskal‐Wallis test for continuous variables and the *χ* ^2^ test or Fisher's exact test for categorical parameters.

All randomized individuals were included in the efficacy and safety analyses, which were conducted according to the intention‐to‐treat principle. The Kaplan–Meier method, along with the log‐rank test, was used to assess the cumulative event risks for the primary outcome within 90 days. To evaluate treatment effects across glycemic status subgroups, Cox proportional hazards regression was applied for time‐to‐event outcomes: new stroke, composite cardiovascular events, ischemic stroke, moderate‐to‐severe bleeding, any bleeding, and all‐cause death, with results expressed as hazard ratios (HRs) and 95% confidence intervals (CIs). For each outcome, only the first event was considered in patients with multiple events, and those event‐free at 90 days were censored at the end of follow‐up or at non‐vascular death. The proportional hazards assumption was confirmed using Schoenfeld residuals. For poor functional outcome and hepatotoxicity at 90 days, log‐binomial regression models were used to estimate relative risks (RRs) with 95% CIs. The interaction of treatment groups with glycemic status subgroups was evaluated by adding interaction terms to the models. Multivariable model (Model 2) was adjusted for covariates comprising age, sex, baseline NIHSS score, baseline mRS, application of lipid‐lowering agents before events, and antiplatelet therapy assignment (clopidogrel‐aspirin and aspirin alone). Model 3 was adjusted for covariates comprising age, sex, baseline NIHSS score, baseline mRS, application of lipid‐lowering agents before events, antiplatelet therapy assignment (clopidogrel‐aspirin and aspirin alone), hypertension, dyslipidemia, and previous ischemic stroke. To examine whether glycemic control could act as an effect modifier for both functional and vascular outcomes, patients with a history of T2DM were further stratified by diabetes duration (diabetes duration < 5 years or diabetes duration ≥ 5 years), and patients with newly diagnosed T2DM or a history of T2DM were further stratified by HbA1c levels (HbA1c level < 7% or HbA1c level ≥ 7%).

All statistical analyses were carried out using SAS statistical software, version 9.4 (SAS Institute), with two‐sided and a *p* value of < 0.05 considered statistically significant.

## 3 Results

### 3.1 Baseline Characteristics

**FIGURE 1:**
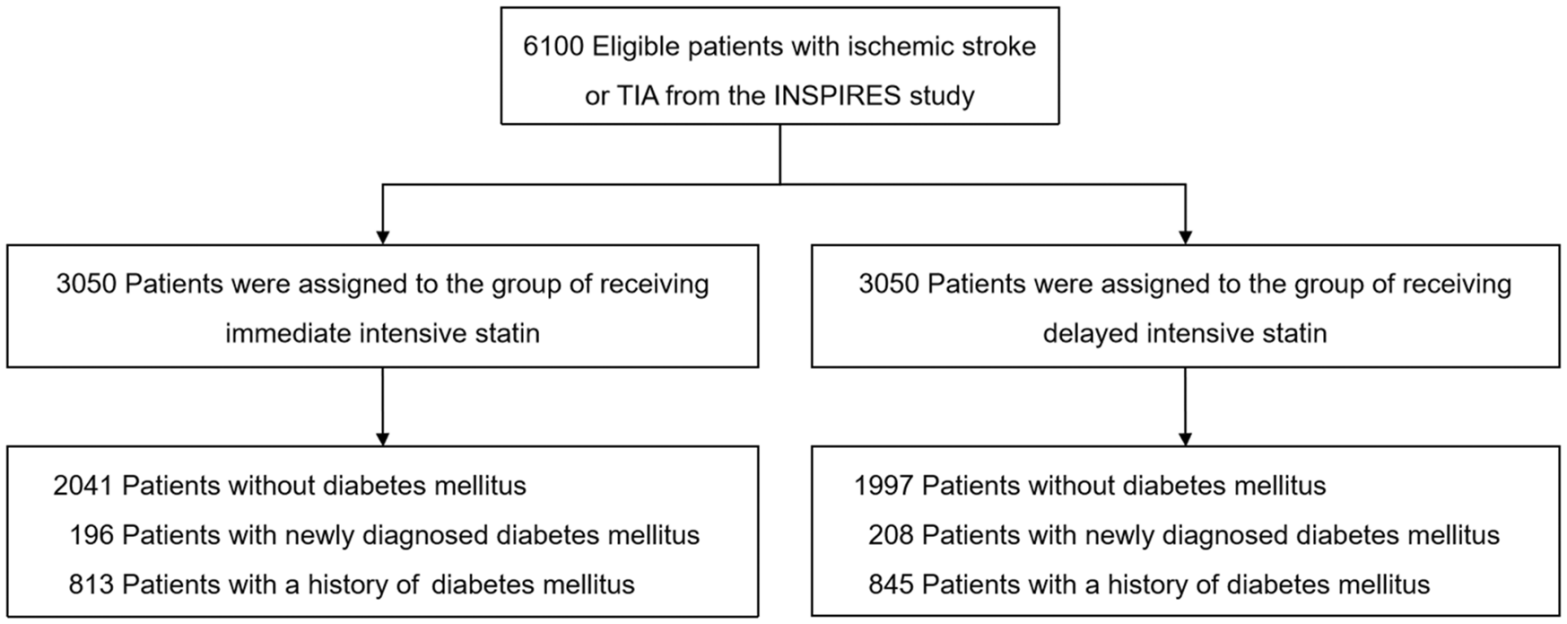
Enrollment and randomization of patients.

A total of 6100 eligible patients were enrolled in the INSPIRES trial over a period spanning September 17, 2018 to October 15, 2022. Randomization yielded 3050 participants in the immediate‐intensive statin group, and an equal number in the delayed‐intensive statin group. The trial included 4038 (66.2%) patients without T2DM (mean age 63.8 ± 9.8 years), 404 (6.6%) patients with a newly diagnosed T2DM (mean age 62.0 ± 10.2 years), and 1658 (27.2%) patients with a history of T2DM (mean age 63.8 ± 9.0 years) (Figure 1). Compared to those without T2DM, patients with a history of T2DM tended to have a higher proportion of females, greater body mass index, and more frequent histories of hypertension, dyslipidemia, and prior ischemic stroke, along with higher use of lipid‐lowering agents, whereas current smoking was less prevalent (Table S1). Baseline characteristics demonstrated a balanced distribution between two intensive statin therapy arms across glycemic status subgroups (Table 1).

**TABLE 1:** Baseline characteristics by glycemic status and statin treatment.

Characteristic	Without T2DM			With newly diagnosed T2DM			With a history of T2DM		
	Delayed statin (*n* = 1997)	Immediate statin (*n* = 2041)	*p*	Delayed statin (*n* = 208)	Immediate statin (*n* = 196)	*p*	Delayed statin (*n* = 845)	Immediate statin (*n* = 813)	*p*
Age, year—Median (IQR)	65 (56–71)	66 (57–72)	0.28	64 (54–69)	64 (56–70)	0.61	65 (58–71)	65 (57–70)	0.12
Female, no. (%)	1344 (67.3)	1424 (69.8)	0.09	125 (60.1)	127 (64.8)	0.33	452 (53.5)	443 (54.5)	0.68
Body‐mass index, kg/m^2^—Median (IQR)	24.2 (22.5–26.2)	24.2 (22.5–26.3)	0.63	24.5 (22.9–27.5)	25.1 (23.3–28.0)	0.34	24.8 (23.0–26.7)	24.9 (23.1–26.9)	0.78
Medical history, no. (%)									
Hypertension	1264 (63.3)	1314 (64.4)	0.47	127 (61.1)	125 (63.8)	0.57	648 (76.7)	605 (74.4)	0.28
Dyslipidemia	53 (2.7)	51 (2.5)	0.76	3 (1.4)	6 (3.1)	0.27	55 (6.5)	58 (7.1)	0.61
Previous ischemic stroke	530 (26.5)	578 (28.3)	0.21	41 (19.7)	52 (26.5)	0.10	311 (36.8)	297 (36.5)	0.91
Previous TIA	30 (1.5)	35 (1.7)	0.59	2 (1.0)	2 (1.0)	0.95	18 (2.1)	10 (1.2)	0.16
Previous myocardial infarction	36 (1.8)	37 (1.8)	0.98	4 (1.92)	2 (1.02)	0.45	20 (2.4)	14 (1.72)	0.35
Current smoker‐no. (%)	639 (32.0)	638 (31.3)	0.61	69 (33.2)	62 (31.6)	0.74	199 (23.6)	176 (21.6)	0.35
Blood pressure, mmHg—Median (IQR)									
Systolic	145 (132–160)	145 (130–160)	0.43	150 (137–167)	150 (135–163)	0.65	146 (134–160)	147 (135–160)	0.47
Diastolic	85 (78–94)	85 (78–94)	0.28	87 (80–98)	88 (80–95)	0.79	83 (77–90)	85 (78–91)	0.06
Application of lipid‐lowering agents before events, no. (%)^a^	149 (7.5)	166 (8.1)	0.43	9 (4.3)	21 (10.7)	0.01	127 (15.0)	115 (14.1)	0.61
Qualifying event, no. (%)									
TIA	245 (12.3)	287 (14.1)	0.09	15 (7.2)	20 (10.2)	0.44	112 (13.3)	122 (15.0)	0.22
Acute single ischemic infarction	349 (17.5)	384 (18.8)		50 (24.0)	40 (20.4)		192 (22.7)	159 (19.6)	
Acute multiple ischemic infarctions	1403 (70.3)	1370 (67.1)		143 (68.8)	136 (69.4)		541 (64.0)	532 (65.4)	
NIHSS in qualifying ischemic stroke, no. (%)^b^									
≤ 3	1352 (77.2)	1344 (76.6)	0.70	149 (77.2)	135 (76.7)	0.91	532 (72.6)	521 (75.4)	0.23
> 3	400 (22.8)	410 (23.4)		44 (22.8)	41 (23.3)		201 (27.4)	170 (24.6)	
ABCD^2^ score among patients with TIA, no. (%)^c^									
≤ 5	225 (91.8)	247 (86.1)	0.04	11 (73.3)	15 (75.0)	0.91	66 (58.9)	77 (63.1)	0.51
> 5	20 (8.2)	40 (13.9)		4 (26.7)	5 (25.0)		46 (41.1)	45 (36.9)	
LDL‐C level at baseline, mmol/L, (IQR)	2.57 (2.11–3.09)	2.44 (2.00–2.95)	< 0.001	2.90 (2.40–3.43)	2.70 (2.28–3.25)	0.049	2.59 (2.10–3.12)	2.54 (1.99–3.16)	0.07
Dual antiplatelet therapy, no. (%)	996 (49.9)	1035 (50.7)	0.60	103 (49.5)	86 (43.9)	0.26	426 (50.4)	404 (49.7)	0.77

Abbreviations: IQR, interquartile range; LDL‐C, low‐density lipoprotein cholesterol; NIHSS, national institutes of health stroke scale; T2DM, type 2 diabetes mellitus; TIA, transient ischemic attack.

^a^ Patients received medication within 1 month before symptom onset.

^b^ Scores on the National Institutes of Health Stroke Scale (NIHSS) range from 0 to 42 for patients with ischemic stroke, with higher scores indicating more severe stroke.

^c^ The ABCD^2^ score assesses the risk of stroke on the basis of age, blood pressure, clinical features, duration of TIA, and the presence or absence of diabetes mellitus for patients with transient ischemic attack, with scores ranging from 0 to 7 and higher scores indicating greater risk.

### 3.2 Efficacy Outcomes

**FIGURE 2:**
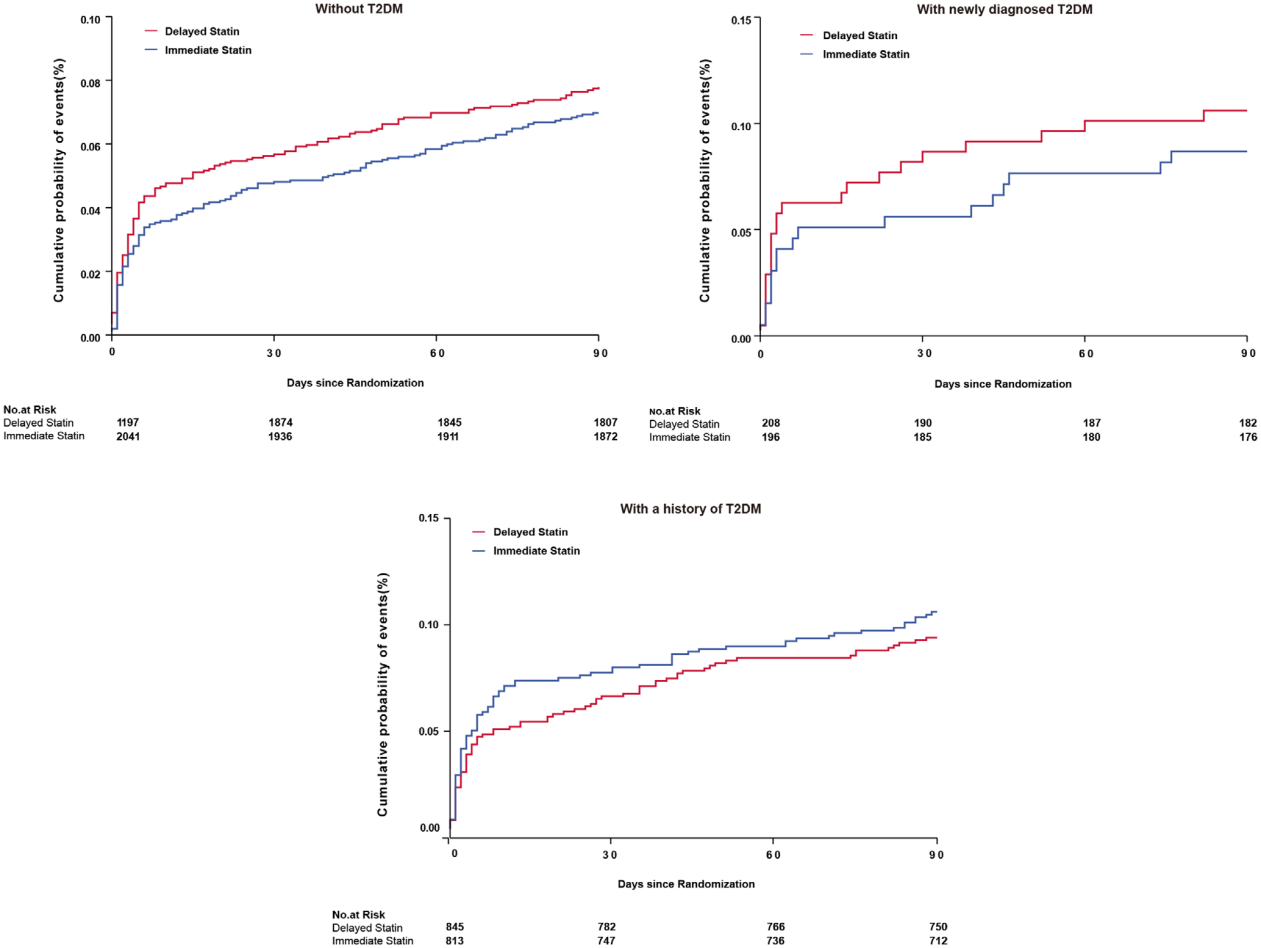
Cumulative probability of stroke in patients with different glycemic status groups and treatments. New stroke in population without type 2 diabetes mellitus (A). New stroke in population with newly diagnosed type 2 diabetes mellitus (B). New stroke in population with a history of type 2 diabetes mellitus (C). HR, hazard ratio; T2DM, type 2 diabetes mellitus.

Compared with patients without T2DM, those with a history of T2DM (HR, 1.35; 95% CI, 1.11–1.64; *p* = 0.003) demonstrated a higher risk of new stroke within 90 days, but not in those newly diagnosed T2DM (HR, 1.26; 95% CI, 0.89–1.78; *p* = 0.19), as reported previously [23]. In the non‐T2DM subgroup, no significant risk reduction (7.1% vs. 7.8%; adjusted HR, 0.89; 95% CI, 0.70–1.12; *p* = 0.31) was found between immediate and delayed statin therapies (Figure 2A). Similarly, in the patients with newly diagnosed T2DM (9.7% vs. 10.6%; adjusted HR, 0.81; 95% CI, 0.42–1.58; *p* = 0.54) and with a history of T2DM (10.6% vs. 9.3%; adjusted HR, 1.19; 95% CI, 0.87–1.64; *p* = 0.27), no obvious risk change between immediate and delayed intensive statin groups (Figure 2B,C). There was no obvious interaction of glycemic status and intensive statin treatment (*p* for interaction = 0.29). Similar trends were observed for both composite vascular event and ischemic stroke (Table 2).

**TABLE 2:** Efficacy and safety outcomes stratified by glycemic status.

Outcome	Immediate statin (*n* = 3050)		Delayed statin (*n* = 3050)		Model 1			Model 2			Model 3		
	Patients, no.	Events, no. (%)	Patients, no.	Events, no. (%)	Unadjusted HR/RR (95% CI)	*p*	*p* for interaction	Adjusted HR/RR (95% CI)	*p*	*p* for interaction	Adjusted HR/RR (95% CI)	*p*	*p* for interaction
Primary outcome													
Stroke^a^													
Without T2DM	2041	142 (7.0)	1997	155 (7.8)	0.89 (0.71–1.12)	0.31	0.38	0.89 (0.70–1.13)	0.33	0.31	0.89 (0.70–1.12)	0.31	0.29
With newly diagnosed T2DM	196	17 (8.7)	208	22 (10.6)	0.81 (0.43–1.52)	0.50		0.79 (0.41–1.53)	0.49		0.81 (0.42–1.58)	0.54	
With a history of T2DM	813	86 (10.6)	845	79 (9.3)	1.14 (0.84–1.54)	0.41		1.19 (0.87–1.63)	0.28		1.19 (0.87–1.64)	0.27	
Secondary outcomes													
Composite vascular event^b^													
Without T2DM	2041	144 (7.1)	1997	156 (7.8)	0.90 (0.71–1.12)	0.34	0.49	0.89 (0.70–1.13)	0.33	0.36	0.89 (0.70–1.12)	0.31	0.34
With newly diagnosed T2DM	196	19 (9.7)	208	22 (10.6)	0.90 (0.49–1.66)	0.74		0.90 (0.47–1.70)	0.74		0.92 (0.49–1.75)	0.80	
With a history of T2DM	813	88 (10.8)	845	82 (9.7)	1.12 (0.83–1.52)	0.45		1.18 (0.86–1.61)	0.30		1.18 (0.87–1.61)	0.29	
Ischemic stroke													
Without T2DM	2041	135 (6.6)	1997	149 (7.5)	0.88 (0.70–1.11)	0.28	0.30	0.88 (0.69–1.12)	0.30	0.25	0.88 (0.69–1.12)	0.29	0.23
With newly diagnosed T2DM	196	16 (8.2)	208	22 (10.6)	0.76 (0.40–1.44)	0.40		0.74 (0.38–1.45)	0.39		0.76 (0.39–1.49)	0.43	
With a history of T2DM	813	84 (10.3)	845	76 (9.0)	1.16 (0.85–1.58)	0.36		1.21 (0.88–1.67)	0.24		1.22 (0.88–1.68)	0.23	
Poor functional outcome^c^													
Without T2DM	2039	158 (7.7)	1994	209 (10.5)	0.74 (0.61–0.90)	0.003	0.03	0.72 (0.57–0.91)	0.005	0.005	0.71 (0.57–0.90)	0.004	0.003
With newly diagnosed T2DM	196	22 (11.2)	207	29 (14.0)	0.80 (0.52–1.23)	0.32		0.64 (0.40–1.01)	0.054		0.66 (0.35–1.22)	0.18	
With a history of T2DM	812	119 (14.7)	845	110 (13.0)	1.13 (0.91–1.40)	0.29		1.17 (0.94–1.45)	0.16		1.18 (0.96–1.46)	0.11	
Primary safety outcomes													
Moderate‐to‐severe bleeding													
Without T2DM	2041	13 (0.6)	1997	12 (0.6)	1.06 (0.48–2.32)	0.89	0.61	1.04 (0.46–2.35)	0.93	0.55	1.03 (0.45–2.34)	0.94	0.54
With newly diagnosed T2DM	196	2 (1.0)	208	1 (0.5)	2.11 (0.19–23.26)	0.54		1.41 (0.11–18.96)	0.79		1.45 (0.11–19.66)	0.78	
With a history of T2DM	813	8 (1.0)	845	4 (0.5)	2.08 (0.63–6.92)	0.23		2.04 (0.61–6.83)	0.25		2.12 (0.63–7.11)	0.23	
Secondary safety outcomes													
Hepatotoxicity													
Without T2DM	2041	29 (1.4)	1997	24 (1.2)	1.18 (0.66–2.13)	0.58	0.90	1.17 (0.65–2.09)	0.60	0.998	1.17 (0.66–2.07)	0.60	0.999
With newly diagnosed T2DM	196	3 (1.5)	208	2 (1.0)	1.59 (0.40–6.30)	0.51		—	—		—	—	
With a history of T2DM	813	7 (0.9)	845	6 (0.7)	1.22 (0.43–3.46)	0.72		1.26 (0.44–3.63)	0.66		1.22 (0.43–3.47)	0.72	
Any bleeding													
Without T2DM	2041	49 (2.4)	1997	55 (2.8)	0.87 (0.59–1.28)	0.47	0.42	0.94 (0.63–1.41)	0.77	0.50	0.94 (0.63–1.40)	0.75	0.50
With newly diagnosed T2DM	196	4 (2.0)	208	7 (3.4)	0.60 (0.18–2.05)	0.60		0.69 (0.19–2.56)	0.58		0.72 (0.19–2.67)	0.62	
With a history of T2DM	813	24 (3.0)	845	18 (2.1)	1.39 (0.76–2.57)	0.29		1.35 (0.73–2.51)	0.34		1.34 (0.72–2.48)	0.36	
All‐cause death													
Without T2DM	2041	17 (0.8)	1997	17 (0.9)	0.98 (0.50–1.92)	0.95	0.64	1.15 (0.55–2.41)	0.71	0.44	1.14 (0.54–2.39)	0.73	0.45
With newly diagnosed T2DM	196	1 (0.5)	208	3 (1.4)	0.35 (0.04–3.37)	0.36		0.26 (0.02–2.78)	0.26		0.24 (0.02–2.74)	0.25	
With a history of T2DM	813	12 (1.5)	845	17 (2.0)	0.73 (0.35–1.53)	0.41		0.80 (0.37–1.75)	0.58		0.85 (0.39–1.87)	0.69	

*Note:* Adjusted HR/RR in Model 2 was adjusted covariates comprising age, sex, baseline NIHSS score, baseline mRS, application of lipid‐lowering agents before events, and antiplatelet therapy assignment (clopidogrel‐aspirin and aspirin alone). Adjusted HR/RR in Model 3 was adjusted covariates comprising age, sex, baseline NIHSS score, baseline mRS, application of lipid‐lowering agents before events, antiplatelet therapy assignment (clopidogrel‐aspirin and aspirin alone), hypertension, dyslipidemia, and previous ischemic stroke. The HRs are shown for stroke, composite cardiovascular events, ischemic stroke, moderate‐to‐severe bleeding, any bleeding, and all‐cause death. The RRs are shown for poor functional outcome (mRS 2–6) and hepatotoxicity.

Abbreviations: HR, hazard ratio; RR, relative risk; T2DM, type 2 diabetes mellitus.

^a^ Includes ischemic and hemorrhagic stroke.

^b^ Includes stroke, myocardial infarction, or death from cardiovascular causes.

^c^ Includes modified Rankin scale scores of 2–6 (range, 0–6, with higher scores indicating more disability and a score of 6 indicating death); data at 90 days were missing in 5 patients in the group of patients without diabetes mellitus, 1 patient in the group of patients with newly diagnosed diabetes mellitus, and 1 patient in the group of patients with a history of diabetes mellitus.

**FIGURE 3:**
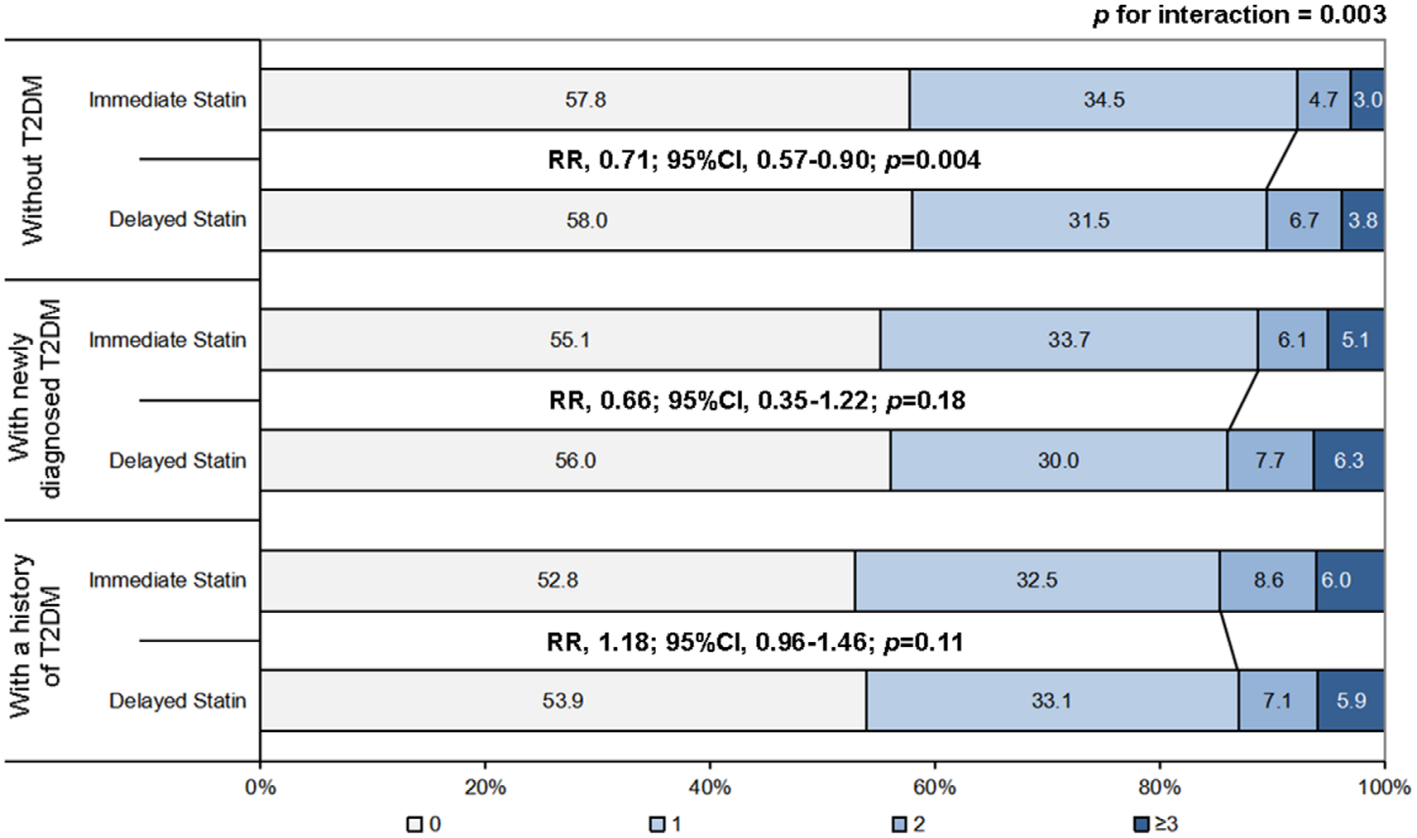
Distribution of mRS score. RR, relative risk; T2DM, type 2 diabetes mellitus.

Notably, a significant treatment‐by‐glycemic status interaction was observed for poor functional outcome risk (*p* for interaction = 0.003) (Table 2). Among patients without T2DM, immediate intensive statin therapy was significantly associated with reduced risk of poor functional outcome at 90 days (7.7% vs. 10.5%; adjusted RR, 0.71; 95% CI, 0.57–0.90; *p* = 0.004). However, no beneficial effect on functional outcome was observed in patients with newly diagnosed T2DM (11.2% vs. 14.0%; adjusted RR, 0.66; 95% CI, 0.35–1.22; *p* = 0.18). No beneficial effect was observed in patients with a history of T2DM (14.7% vs. 13.0%; adjusted RR, 1.18; 95% CI, 0.96–1.46; *p* = 0.11) (Figure 3).

### 3.3 Safety Outcomes

No significant differences in risk of safety outcomes were observed between intensive statin therapy groups across glycemic status subgroups (Table 2). Within 90 days, immediate intensive statin therapy showed no increased risk of moderate‐to‐severe bleeding in patients without T2DM (0.6% vs. 0.6%; adjusted HR, 1.03; 95% CI, 0.45–2.34; *p* = 0.94), in patients with newly diagnosed T2DM (1.0% vs. 0.5%; adjusted HR, 1.45; 95% CI, 0.11–19.66; *p* = 0.78), or in patients with a history of T2DM (1.0% vs. 0.5%; adjusted HR, 2.12; 95% CI, 0.63–7.11; *p* = 0.23) (*p* for interaction = 0.54). This safety profile remained consistent for hepatotoxicity, any bleeding events, and all‐cause mortality across all subgroups. Immediate intensive statin therapy demonstrated comparable tolerability profiles across glycemic status subgroups.

To examine whether glycemic control could act as an effect modifier for both functional and vascular outcomes, patients with newly diagnosed T2DM or a history of T2DM were further stratified by HbA1c levels (HbA1c level < 7% or HbA1c level ≥ 7%) (Tables S2 and S3); patients with a history of T2DM were further stratified by diabetes duration (diabetes duration < 5 years or diabetes duration ≥ 5 years) (Table S4). No interaction of treatment and glycemic control (HbA1c level or diabetes duration) was observed. However, due to the limited sample size within each subgroup, the results regarding the absence of interaction should be interpreted with caution.

## 4 Discussion

In this prespecified subgroup analysis, no substantial differences were observed in the risk of new strokes and composite vascular events with immediate‐intensive statin treatment across patients with glycemic status. However, a functional benefit emerged among patients without T2DM, who showed a lower risk of poor outcome with immediate‐intensive therapy. This observed effect of immediate‐intensive statin therapy appeared to differ across glycemic subgroups, with no significant risk reduction identified in patients with newly diagnosed T2DM or a history of T2DM. Furthermore, safety outcomes, including bleeding, hepatotoxicity, and all‐cause death, showed no between‐group differences regardless of glycemic status. These findings reinforce the potential benefit of initiating immediate intensive statin therapy within 72 h of symptom onset, particularly in patients without glucose metabolism abnormalities.

In diabetic patients, post‐stroke management is complicated by the close interaction between metabolic abnormalities and cerebrovascular risk, necessitating more nuanced therapeutic approaches. Abnormal glucose metabolism is highly prevalent among stroke survivors, with diabetes mellitus (a key manifestation of glucose dysregulation) recognized as an independent risk factor for stroke recurrence, contributing to a 56% increase in relative mortality risk following ischemic stroke and TIA [28, 29, 30]. High‐intensive statin therapy has become a cornerstone of contemporary stroke management, as recommended by current guidelines [6]. Growing evidence highlights the modulatory role of glycemic status in statin efficacy during vascular disease recovery [31]. The SPARCL trial subgroup analysis revealed a reduced impact of atorvastatin on stroke recurrence, showing similar benefits across both diabetic and non‐diabetic cohorts [19]. Whereas findings from the Evaluation of Cardiovascular Outcomes After an Acute Coronary Syndrome During Treatment With Alirocumab (ODYSSEY OUTCOMES) study demonstrated that lipid‐lowering therapy yielded nearly twice the absolute reduction in cardiovascular events among patients with diabetes compared to those without diabetes [32]. Furthermore, the Improved Reduction of Outcomes Vytorin Efficacy International Trial (IMPROVE‐IT) indicated that the addition of ezetimibe to statin provided greater benefits in patients with diabetes [33]. However, our study suggested that the beneficial effects of early intensive statin therapy on functional outcomes may be more pronounced in patients without T2DM, compared to individuals with diabetes. This finding underscores the importance of personalized treatment strategies in stroke management, particularly in patients with T2DM.

This attenuated response to statin therapy in patients with diabetes mellitus (both newly diagnosed and established) may be attributed to the cumulative vascular injury induced by chronic hyperglycemia. Prolonged exposure to elevated glucose levels promotes the accumulation of advanced glycation end‐products (AGEs) [34, 35, 36], exacerbates microvascular dysfunction [37, 38], and perpetuates a state of chronic systemic inflammation [39, 40]. These pathophysiological changes likely compromise vascular reparative capacity, diminishing the cerebrovascular benefits of statins. Previous reports indicated early glycemic control could confer more benefits on vascular function compared to interventions initiated after prolonged metabolic dysregulation [41, 42]. Our data suggested that even patients with newly diagnosed T2DM exhibit reduced statin efficacy. This implied that significant vascular damage may predate clinical diagnosis, limiting therapeutic responsiveness. Moreover, increasing evidence indicated that statins can impair insulin signaling pathways and disrupt glycemic control [43, 44]. Long‐term or intensive statin use was associated with an increased risk of diabetes and higher fasting blood glucose [45, 46]. In diabetic mice, statins may increase insulin resistance, disrupt lipid metabolism, promote inflammation, and fuel diabetic nephropathy progression [47]. This suggested that using statins in diabetic patients might worsen glycemic control and accelerate complications, potentially counteracting their intended benefits.

Consequently, the lack of functional benefit observed in T2DM patients implies that chronic hyperglycemia may contribute to persistent vascular changes that influence the response to statin therapy. These findings highlight the need for a precision medicine approach in stroke management. Alternative lipid‐lowering strategies may need to be explored for individuals with established diabetes to optimize both vascular protection and functional recovery.

Several limitations that should be acknowledged. First, the subgroup of patients with newly diagnosed T2DM, a key subgroup in this analysis, had the smallest sample size, and information on diabetes duration was not available. This limited our ability to perform more detailed analyses and warrants cautious interpretation of findings within this subgroup. Additionally, the classification of glycemic status (e.g., newly diagnosed vs. a history of T2DM) may not fully capture heterogeneity within each group, as the newly diagnosed T2DM group may include individuals with undetected long‐term hyperglycemia, whereas those with known T2DM vary widely in disease duration and glycemic control. Although additional analyses showed no interaction between statin treatment and glycemic parameters (HbA1c/diabetes duration), these results should be interpreted cautiously due to the aforementioned limitation. Furthermore, residual confounding from unmeasured factors, such as glycemic variability, might partially explain the observed effect modification. Third, the limited sample size within each glycemic subgroup, particularly the subgroup with newly diagnosed T2DM, combined with the low proportion of female participants may reduce the statistical power to detect differences across glycemic status groups. Finally, in this subgroup analysis, the primary endpoint of stroke recurrence showed no significant reduction across glycemic subgroups, the observed divergence in functional outcomes may reflect distinct biological mechanisms. Statin‐mediated neuroprotection, through pleiotropic effects on endothelial function, inflammation modulation, and neural repair pathways, could disproportionately influence functional recovery than on stroke recurrence prevention, particularly in populations with preserved metabolic plasticity. However, the proposed mechanisms remain hypothetical and require further validation in future studies.

## 5 Conclusion

This analysis demonstrated that immediate intensive statin therapy improved functional outcomes in patients without T2DM, whereas no such association was observed in those with newly diagnosed or pre‐existing T2DM, and no differences in safety outcomes were identified across subgroups. These findings support the use of immediate‐intensive statin in the acute phase, particularly for normoglycemic populations, and highlight the need for individualized treatment strategies in patients with established T2DM.

## Author Contributions

Y.W. contributed to the conception and design of the study; L.J., Z.D., Y.G., X.W., Y.Y., T.W., Y.P., W.C., and Y.W. contributed to acquisition and analysis of data; L.J. and Z.D. contributed to drafting the manuscript. L.J. and X.W. contributed to preparing the figures; Y.P., W.C., and Y.W. reviewed of the manuscript for important intellectual content. All authors have full access to all data used in the study and take responsibility for the integrity of the data and the accuracy of the data analysis. All authors have read and agreed to the published version of the manuscript.

## Funding

The study was supported by grants from the National Natural Science Foundation of China (nos. 82425101 and 82101358), Beijing Nova Program (20230484245), Capital's Funds for Health Improvement and Research (2022‐2‐2045), the National Key R&D Program of China (nos. 2022YFF1501500, 2022YFF1501501, 2022YFF1501502, 2022YFF1501503, 2022YFF1501504, 2022YFF1501505, 2017YFC1307900 and 2017YFC1307905), Beijing Laboratory of Oral Health (PXM2021_014226_000041), Sanofi, and Jialin Pharmaceutical. The funders of the study had no role in the study design, data collection, data analysis, data interpretation, or writing of the report.

## Ethics Statement

The Ethics Committees at Beijing Tiantan Hospital (Review Board approval number: KY2017‐065‐02) and at each participating center approved this trial.

## Conflicts of Interest

The authors declare no conflicts of interest.

## Supporting information


**Table S1:** cns70082‐sup‐0001‐TablesS1‐S4.docx.
**Table S2:** cns70082‐sup‐0001‐TablesS1‐S4.docx.
**Table S3:** cns70082‐sup‐0001‐TablesS1‐S4.docx.
**Table S4:** cns70082‐sup‐0001‐TablesS1‐S4.docx.

## Data Availability

The original contributions presented in the study are included in the article; further inquiries can be directed to the corresponding authors.
