# Supplementary material for: Glycemic Status and Effect of Immediate Intensive Statin on Mild Ischemic Stroke: A Subgroup Analysis of the INSPIRES Trial
Source: CNS Neurosci Ther. 2026 Apr 27;32(4):e70882. doi: 10.1002/cns.70882 (PMC13112190; doi:10.1002/cns.70882)
Supplement: Supplementary file 1 — Table S1: cns70082‐sup‐0001‐TablesS1‐S4.docx. Table S2: cns70082‐sup‐0001‐TablesS1‐S4.docx. Table S3: cns70082‐sup‐0001‐TablesS1‐S4.docx. Table S4: cns70082‐sup‐0001‐TablesS1‐S4.docx. [file CNS-32-e70882-s001.docx]

### **SUPPLEMENT MATERIALS**

1. Table S1. Baseline characteristics of participants according to glycemic status.
2. Table S2 Efficacy and safety outcomes stratified by HbA1c level in patients with newly diagnosed T2DM.
3. Table S3 Efficacy and safety outcomes stratified by HbA1c level in patients with a history of T2DM.
4. Table S4 Efficacy and safety outcomes stratified by diabetes duration in patients with a history of T2DM.

**Table S1. Baseline characteristics of participants according to glycemic status.**

| **Characteristic** | **Without** **T2DM**  **(n=4038)** | **With newly diagnosed T2DM**  **(n=404)** | **With a history of T2DM**  **(n=1658)** | ***p* value** |
| --- | --- | --- | --- | --- |
| Age, yr-Median (IQR) | 65(57-72) | 64(55-69) | 65(57-70) | 0.004 |
| Female-No. (%) | 1270(31.5) | 152(37.6) | 763(46.0) | <0.001 |
| Body-mass index, kg/m^2^-Median (IQR) | 24.2(22.5-26.3) | 24.8(23.0-27.8) | 24.8(23.0-26.8) | <0.001 |
| Medical history-No. (%) |  |  |  |  |
| Hypertension | 2578(63.8) | 252(62.4) | 1253(75.6) | <0.001 |
| Dyslipidemia | 104(2.6) | 9(2.2) | 113(6.8) | <0.001 |
| Previous ischemic stroke | 1108(27.4) | 93(23.0) | 608(36.7) | <0.001 |
| Previous TIA | 65(1.6) | 4(1.0) | 28(1.7) | 0.59 |
| Previous myocardial infarction | 73(1.8) | 6(1.5) | 34(2.1) | 0.70 |
| Current smoker-No. (%) | 1277(31.6) | 131(32.4) | 375(22.6) | <0.001 |
| Blood Pressure, mmHg-Median (IQR) |  |  |  |  |
| Systolic | 145(132-160) | 150(136-165) | 147(134-160) | <0.001 |
| Diastolic | 85(78-94) | 88(80-97) | 83(78-90) | <0.001 |
| Application of lipid-lowering agents before events-No. (%) † | 315(7.8) | 30(7.4) | 242(14.6) | <0.001 |
| Qualifying event-No. (%) |  |  |  | 0.001 |
| TIA | 532(13.2) | 35(8.7) | 234(14.1) |  |
| Acute single ischemic infarction | 733(18.2) | 90(22.3) | 351(21.2) |  |
| Acute multiple ischemic infarctions | 2773(68.7) | 279(69.1) | 1073(64.7) |  |
| NIHSS in qualifying ischemic stroke, no. (%) # |  |  |  | 0.08 |
| ≤3 | 2696(76.9) | 284(77.0) | 1053(73.9) |  |
| >3 | 810(23.1) | 85(23.0) | 371(26.1) |  |
| ABCD^2^ score among patients with TIA, no. (%)∥ |  |  |  | <0.001 |
| ≤5 | 472(88.7) | 26(74.3) | 143(61.1) |  |
| >5 | 60(11.3) | 9(25.7) | 91(38.9) |  |
| LDL-C level at baseline, mmol/L, (IQR) | 2.51(2.05-3.03) | 2.84(2.34-3.34) | 2.56(2.05-3.14) | <0.001 |
| Dual antiplatelet therapy-No. (%) | 2031(50.3) | 189(46.8) | 830(50.1) | 0.40 |

Abbreviations: T2DM, type 2 diabetes mellitus; IQR, interquartile range; TIA, transient ischemic attack; NIHSS, national institutes of health stroke scale; LDL-C, low-density lipoprotein cholesterol.

† Patients received medication within 1 month before symptom onset.

# Scores on the National Institutes of Health Stroke Scale (NIHSS) range from 0 to 42 for patients with ischemic stroke, with higher scores indicating more severe stroke.

∥ The ABCD^2^ score assesses the risk of stroke on the basis of age, blood pressure, clinical features, duration of TIA, and the presence or absence of diabetes mellitus for patients with transient ischemic attack, with scores ranging from 0 to 7 and higher scores indicating greater risk.

### TABLE S2 Efficacy and safety outcomes stratified by HbA1c level in patients with newly diagnosed T2DM.

| **Outcome** | **Immediate Statin (n=185)** | | **Delayed Statin**  **(n=192)** | | **Model 1** | | | **Model 2** | | | **Model 3** | | |
| --- | --- | --- | --- | --- | --- | --- | --- | --- | --- | --- | --- | --- | --- |
|  | **Patients, No.** | **Events, No.(%)** | **Patients, No.** | **Events, No.(%)** | **Unadjusted**  **HR/RR (95%CI)** | ***p* value** | ***p* for interaction** | **Adjusted HR/RR**  **(95%CI)** | ***p* value** | ***p* for interaction** | **Adjusted HR/RR**  **(95%CI)** | ***p* value** | ***p* for interaction** |
| **Primary outcome** |  |  |  |  |  |  |  |  |  |  |  |  |  |
| Stroke* |  |  |  |  |  |  | 0.54 |  |  | 0.79 |  |  | 0.83 |
| HbA1c level <7% | 52 | 4(7.7) | 58 | 8(13.8) | 0.54(0.16-1.81) | 0.32 |  | 1.05(0.28-3.97) | 0.95 |  | 1.09(0.28-4.29) | 0.90 |  |
| HbA1c level ≥7% | 133 | 12(9.0) | 134 | 14(10.4) | 0.85(0.39-1.83) | 0.67 |  | 0.67(0.29-1.53) | 0.34 |  | 0.75(0.33-1.69) | 0.48 |  |
| **Secondary outcomes** |  |  |  |  |  |  |  |  |  |  |  |  |  |
| Composite vascular event# |  |  |  |  |  |  | 0.40 |  |  | 0.93 |  |  | 0.96 |
| HbA1c level <7% | 52 | 4(7.7) | 58 | 8(13.8) | 0.54(0.16-1.81) | 0.32 |  | 1.05(0.28-3.97) | 0.95 |  | 1.09(0.28-4.29) | 0.90 |  |
| HbA1c level ≥7% | 133 | 14(10.5) | 134 | 14(10.4) | 0.99(0.47-2.08) | 0.98 |  | 0.87(0.40-1.90) | 0.73 |  | 0.86(0.39-1.89) | 0.70 |  |
| Ischemic stroke |  |  |  |  |  |  | 0.63 |  |  | 0.70 |  |  | 0.75 |
| HbA1c level <7% | 52 | 4(7.7) | 58 | 8(13.8) | 0.54(0.16-1.81) | 0.32 |  | 1.05(0.28-3.97) | 0.95 |  | 1.09(0.28-4.29) | 0.90 |  |
| HbA1c level ≥7% | 133 | 11(8.3) | 134 | 14(10.4) | 0.78(0.35-1.71) | 0.53 |  | 0.70(0.31-1.59) | 0.39 |  | 0.70(0.30-1.60) | 0.39 |  |
| Poor functional outcome† |  |  |  |  |  |  | 0.55 |  |  | 0.97 |  |  | 0.93 |
| HbA1c level <7% | 52 | 3(5.8) | 57 | 6(10.5) | 0.55(0.19-1.60) | 0.27 |  | 0.65(0.23-1.88) | 0.43 |  | - | - |  |
| HbA1c level ≥7% | 133 | 18(13.5) | 134 | 21(15.7) | 0.86(0.52-1.44) | 0.57 |  | 0.69(0.39-1.21) | 0.19 |  | - | - |  |
| **Primary safety outcomes** |  |  |  |  |  |  |  |  |  |  |  |  |  |
| Moderate-to-severe bleeding |  |  |  |  |  |  | 1.00 |  |  | 1.00 |  |  | 1.00 |
| HbA1c level <7% | 52 | 1(1.9) | 58 | 0(0.0) | - | - |  | - | - |  | - | - |  |
| HbA1c level ≥7% | 133 | 1(0.8) | 134 | 1(0.7) | 1.00(0.06-15.99) | 1.00 |  | 0.38(0.01-21.91) | 0.64 |  | 0.16(0.00-41.44) | 0.51 |  |
| **Secondary safety outcomes** |  |  |  |  |  |  |  |  |  |  |  |  |  |
| Hepatotoxicity |  |  |  |  |  |  | 1.00 |  |  | 0.92 |  |  | 0.91 |
| HbA1c level <7% | 52 | 0(0.0) | 58 | 1(1.7) | - | - |  | - | - |  | - | - |  |
| HbA1c level ≥7% | 133 | 3(2.3) | 134 | 0(0.0) | - | - |  | - | - |  | - | - |  |
| Any bleeding |  |  |  |  |  |  | 0.90 |  |  | 0.62 |  |  | 1.00 |
| HbA1c level <7% | 52 | 1(1.9) | 58 | 2(3.4) | 0.55(0.05-6.04) | 0.62 |  | - | - |  | - | - |  |
| HbA1c level ≥7% | 133 | 2(1.5) | 134 | 3(2.2) | 0.67(0.11-3.99) | 0.66 |  | 0.41(0.06-3.02) | 0.38 |  | 0.31(0.02-4.37) | 0.39 |  |
| All-cause death |  |  |  |  |  |  | 1.00 |  |  | 1.00 |  |  | 0.53 |
| HbA1c level <7% | 52 | 0(0.0) | 58 | 1(1.7) | - | - |  | - | - |  | - | - |  |
| HbA1c level ≥7% | 133 | 1(0.8) | 134 | 2(1.5) | 0.50(0.05-5.50) | 0.57 |  | 0.40(0.03-4.78) | 0.47 |  | 0.19(0.02-2.25) | 0.19 |  |

Note: Adjusted HR/RR in model 2 was adjusted covariates comprising age, sex, baseline NIHSS score, baseline mRS, application of lipid-lowering agents before events, and antiplatelet therapy assignment (clopidogrel-aspirin and aspirin alone). Adjusted HR/RR in model 3 was adjusted covariates comprising age, sex, baseline NIHSS score, baseline mRS, application of lipid-lowering agents before events, antiplatelet therapy assignment (clopidogrel-aspirin and aspirin alone), hypertension, dyslipidemia, and previous ischemic stroke. The HRs are shown for stroke, composite cardiovascular events, ischemic stroke, moderate-to-severe bleeding, any bleeding, and all-cause death. The RRs are shown for poor functional outcome (mRS 2-6) and hepatotoxicity.

* Includes ischemic and hemorrhagic stroke.

# Includes stroke,myocardial infarction, or death from cardiovascular causes.

† Includes modified Rankin scale scores of 2 to 6 (range, 0 to 6, with higher scores indicating more disability and a score of 6 indicating death); data at 90 days were missing in 5 patients in the group of patients without diabetes mellitus, 1 patients in the group of patients with newly diagnosed diabetes mellitus, and 1 patients in the group of patients with a history of diabetes mellitus.

Abbreviations: HR, hazard ratio; RR, relative risk; T2DM, type 2 diabetes mellitus.

### TABLE S3 Efficacy and safety outcomes stratified by HbA1c level in patients with a history of T2DM.

| **Outcome** | **Immediate Statin (n=753)** | | **Delayed Statin**  **(n=813)** | | **Model 1** | | | **Model 2** | | | **Model 3** | | |
| --- | --- | --- | --- | --- | --- | --- | --- | --- | --- | --- | --- | --- | --- |
|  | **Patients, No.** | **Events, No.(%)** | **Patients, No.** | **Events, No.(%)** | **Unadjusted**  **HR/RR (95%CI)** | ***p* value** | ***p* for interaction** | **Adjusted HR/RR**  **(95%CI)** | ***p* value** | ***p* for interaction** | **Adjusted HR/RR**  **(95%CI)** | ***p* value** | ***p* for interaction** |
| **Primary outcome** |  |  |  |  |  |  |  |  |  |  |  |  |  |
| Stroke* |  |  |  |  |  |  | 0.65 |  |  | 0.81 |  |  | 0.81 |
| HbA1c level <7% | 173 | 9(5.2) | 180 | 10(5.6) | 0.94(0.38-2.30) | 0.88 |  | 1.03(0.40-2.66) | 0.95 |  | 1.11(0.43-2.88) | 0.83 |  |
| HbA1c level ≥7% | 580 | 66(11.4) | 633 | 62(9.8) | 1.17(0.83-1.66) | 0.37 |  | 1.13(0.79-1.63) | 0.50 |  | 1.21(0.84-1.73) | 0.31 |  |
| **Secondary outcomes** |  |  |  |  |  |  |  |  |  |  |  |  |  |
| Composite vascular event# |  |  |  |  |  |  | 0.51 |  |  | 0.64 |  |  | 0.65 |
| HbA1c level <7% | 173 | 9(5.2) | 180 | 11(6.1) | 0.85(0.35-2.05) | 0.72 |  | 0.91(0.36-2.28) | 0.84 |  | 0.97(0.38-2.44) | 0.94 |  |
| HbA1c level ≥7% | 580 | 68(11.7) | 633 | 64(10.1) | 1.17(0.83-1.65) | 0.37 |  | 1.21(0.85-1.72) | 0.30 |  | 1.21(0.85-1.72) | 0.29 |  |
| Ischemic stroke |  |  |  |  |  |  | 0.46 |  |  | 0.61 |  |  | 0.61 |
| HbA1c level <7% | 173 | 8(4.6) | 180 | 10(5.6) | 0.83(0.33-2.11) | 0.70 |  | 0.94(0.36-2.50) | 0.91 |  | 1.03(0.39-2.74) | 0.96 |  |
| HbA1c level ≥7% | 580 | 65(11.2) | 633 | 59(9.3) | 1.21(0.85-1.72) | 0.28 |  | 1.25(0.87-1.80) | 0.24 |  | 1.25(0.87-1.80) | 0.23 |  |
| Poor functional outcome† |  |  |  |  |  |  | 0.82 |  |  | 0.81 |  |  | 0.84 |
| HbA1c level <7% | 173 | 21(12.1) | 180 | 18(10.0) | 1.21(0.71-2.07) | 0.48 |  | 1.16(0.69-1.94) | 0.59 |  | 1.18(0.68-2.02) | 0.56 |  |
| HbA1c level ≥7% | 579 | 85(14.7) | 633 | 83(13.1) | 1.12(0.88-1.43) | 0.36 |  | 1.26(0.95-1.68) | 0.11 |  | 1.27(0.96-1.69) | 0.10 |  |
| **Primary safety outcomes** |  |  |  |  |  |  |  |  |  |  |  |  |  |
| Moderate-to-severe bleeding |  |  |  |  |  |  | 0.99 |  |  | 1.00 |  |  | 1.00 |
| HbA1c level <7% | 173 | 1(0.6) | 180 | 0(0.0) | - | - |  | - | - |  | - | - |  |
| HbA1c level ≥7% | 580 | 7(1.2) | 633 | 4(0.6) | 1.92(0.56-6.56) | 0.30 |  | 1.83(0.53-6.34) | 0.34 |  | 1.90(0.54-6.60) | 0.32 |  |
| **Secondary safety outcomes** |  |  |  |  |  |  |  |  |  |  |  |  |  |
| Hepatotoxicity |  |  |  |  |  |  | 0.82 |  |  | 0.86 |  |  | 0.79 |
| HbA1c level <7% | 173 | 1(0.6) | 180 | 1(0.6) | 1.04(0.07-15.42) | 0.98 |  | 0.69(0.08-6.28) | 0.74 |  | - | - |  |
| HbA1c level ≥7% | 580 | 6(1.0) | 633 | 5(0.8) | 1.31(0.43-3.98) | 0.63 |  | 1.37(0.45-4.19) | 0.58 |  | 1.39(0.46-4.20) | 0.56 |  |
| Any bleeding |  |  |  |  |  |  | 0.27 |  |  | 0.26 |  |  |  |
| HbA1c level <7% | 173 | 4(2.3) | 180 | 1(0.6) | 4.2(0.47-37.51) | 0.20 |  | 6.10(0.61-61.13) | 0.12 |  |  |  |  |
| HbA1c level ≥7% | 580 | 17(2.9) | 633 | 16(2.5) | 1.17(0.59-2.31) | 0.66 |  | 1.13(0.57-2.27) | 0.72 |  |  |  |  |
| All-cause death |  |  |  |  |  |  | 0.84 |  |  | 0.89 |  |  | 0.85 |
| HbA1c level <7% | 173 | 2(1.2) | 180 | 2(1.1) | 1.04(0.15-7.37) | 0.97 |  | 0.83(0.11-6.36) | 0.86 |  | 0.97(0.12-7.79) | 0.98 |  |
| HbA1c level ≥7% | 580 | 10(1.7) | 633 | 13(2.1) | 0.84(0.37-1.92) | 0.68 |  | 0.91(0.38-2.19) | 0.84 |  | 0.96(0.40-2.32) | 0.93 |  |

Note: Adjusted HR/RR in model 2 was adjusted covariates comprising age, sex, baseline NIHSS score, baseline mRS, application of lipid-lowering agents before events, and antiplatelet therapy assignment (clopidogrel-aspirin and aspirin alone). Adjusted HR/RR in model 3 was adjusted covariates comprising age, sex, baseline NIHSS score, baseline mRS, application of lipid-lowering agents before events, antiplatelet therapy assignment (clopidogrel-aspirin and aspirin alone), hypertension, dyslipidemia, and previous ischemic stroke. The HRs are shown for stroke, composite cardiovascular events, ischemic stroke, moderate-to-severe bleeding, any bleeding, and all-cause death. The RRs are shown for poor functional outcome (mRS 2-6) and hepatotoxicity.

* Includes ischemic and hemorrhagic stroke.

# Includes stroke,myocardial infarction, or death from cardiovascular causes.

† Includes modified Rankin scale scores of 2 to 6 (range, 0 to 6, with higher scores indicating more disability and a score of 6 indicating death); data at 90 days were missing in 5 patients in the group of patients without diabetes mellitus, 1 patients in the group of patients with newly diagnosed diabetes mellitus, and 1 patients in the group of patients with a history of diabetes mellitus.

Abbreviations: HR, hazard ratio; RR, relative risk; T2DM, type 2 diabetes mellitus.

### TABLE S4 Efficacy and safety outcomes stratified by diabetes duration in patients with a history of T2DM.

| **Outcome** | **Immediate Statin (n=797)** | | **Delayed Statin**  **(n=833)** | | **Model 1** | | | **Model 2** | | | **Model 3** | | |
| --- | --- | --- | --- | --- | --- | --- | --- | --- | --- | --- | --- | --- | --- |
|  | **Patients, No.** | **Events, No.(%)** | **Patients, No.** | **Events, No.(%)** | **Unadjusted**  **HR/RR (95%CI)** | ***p* value** | ***p* for interaction** | **Adjusted HR/RR**  **(95%CI)** | ***p* value** | ***p* for interaction** | **Adjusted HR/RR**  **(95%CI)** | ***p* value** | ***p* for interaction** |
| **Primary outcome** |  |  |  |  |  |  |  |  |  |  |  |  |  |
| Stroke* |  |  |  |  |  |  | 0.06 |  |  | 0.11 |  |  | 0.11 |
| diabetes duration <5 years | 284 | 36(12.7) | 290 | 22(7.6) | 1.71(1.01-2.90) | 0.048 |  | 1.74(0.99-3.07) | 0.06 |  | 1.73(0.98-3.06) | 0.06 |  |
| diabetes duration ≥5 years | 513 | 50(9.7) | 543 | 57(10.5) | 0.92(0.63-1.35) | 0.68 |  | 1.01(0.68-1.49) | 0.96 |  | 1.02(0.69-1.50) | 0.94 |  |
| **Secondary outcomes** |  |  |  |  |  |  |  |  |  |  |  |  |  |
| Composite vascular event# |  |  |  |  |  |  | 0.04 |  |  | 0.07 |  |  | 0.07 |
| diabetes duration <5 years | 284 | 37(13.0) | 290 | 22(7.6) | 1.76(1.04-2.98) | 0.04 |  | 1.79(1.02-3.15) | 0.04 |  | 1.78(1.01-3.13) | 0.046 |  |
| diabetes duration ≥5 years | 513 | 51(9.9) | 543 | 60(11.0) | 0.90(0.62-1.30) | 0.56 |  | 0.99(0.67-1.44) | 0.94 |  | 0.99(0.68-1.45) | 0.96 |  |
| Ischemic stroke |  |  |  |  |  |  | 0.09 |  |  | 0.15 |  |  | 0.15 |
| diabetes duration <5 years | 284 | 34(12.0) | 290 | 21(7.2) | 1.69(0.98-2.91) | 0.06 |  | 1.74(0.97-3.12) | 0.06 |  | 1.72(0.96-3.08) | 0.07 |  |
| diabetes duration ≥5 years | 513 | 50(9.7) | 543 | 55(10.1) | 0.96(0.65-1.41) | 0.83 |  | 1.05(0.71-1.55) | 0.81 |  | 1.06(0.71-1.56) | 0.79 |  |
| Poor functional outcome† |  |  |  |  |  |  | 0.53 |  |  | 0.91 |  |  | 0.96 |
| diabetes duration <5 years | 284 | 44(15.5) | 290 | 36(12.4) | 1.25(0.91-1.72) | 0.17 |  | 1.17(0.84-1.65) | 0.35 |  | 1.17(0.83-1.64) | 0.37 |  |
| diabetes duration ≥5 years | 512 | 74(14.5) | 543 | 74(13.6) | 1.06(0.80-1.40) | 0.68 |  | 1.18(0.93-1.62) | 0.18 |  | 1.22(0.95-1.55) | 0.12 |  |
| **Primary safety outcomes** |  |  |  |  |  |  |  |  |  |  |  |  |  |
| Moderate-to-severe bleeding |  |  |  |  |  |  | 0.25 |  |  | 0.28 |  |  | 0.31 |
| diabetes duration <5 years | 284 | 5(1.8) | 290 | 1(0.3) | 5.15(0.60-44.04) | 0.13 |  | 5.26(0.61-45.53) | 0.13 |  | 6.26(0.70-56.15) | 0.10 |  |
| diabetes duration ≥5 years | 513 | 50(9.7) | 543 | 55(10.1) | 1.06(0.21-5.23) | 0.95 |  | 0.98(0.19-5.08) | 0.98 |  | 1.11(0.21-6.00) | 0.90 |  |
| **Secondary safety outcomes** |  |  |  |  |  |  |  |  |  |  |  |  |  |
| Hepatotoxicity |  |  |  |  |  |  | 0.35 |  |  | 0.40 |  |  | 0.51 |
| diabetes duration <5 years | 284 | 1(0.4) | 290 | 2(0.7) | 0.51(0.05-5.33) | 0.57 |  | - | - |  | - | - |  |
| diabetes duration ≥5 years | 513 | 6(1.2) | 543 | 4(0.7) | 1.59(0.37-6.88) | 0.54 |  | 1.66(0.41-6.66) | 0.48 |  | 1.56(0.39-6.20) | 0.53 |  |
| Any bleeding |  |  |  |  |  |  | 0.30 |  |  | 0.31 |  |  | 0.33 |
| diabetes duration <5 years | 284 | 9(3.2) | 290 | 9(3.1) | 1.03(0.41-2.59) | 0.95 |  | 1.00(0.40-2.55) | 0.99 |  | 1.05(0.41-2.67) | 0.92 |  |
| diabetes duration ≥5 years | 513 | 15(2.9) | 543 | 8(1.5) | 2.00(0.85-4.71) | 0.11 |  | 1.93(0.81-4.62) | 0.14 |  | 1.87(0.78-4.50) | 0.16 |  |
| All-cause death |  |  |  |  |  |  | 0.52 |  |  | 0.69 |  |  | 0.76 |
| diabetes duration <5 years | 284 | 5(1.8) | 290 | 5(1.7) | 1.03(0.30-3.54) | 0.97 |  | 0.97(0.24-3.93) | 0.97 |  | 0.96(0.24-3.92) | 0.96 |  |
| diabetes duration ≥5 years | 513 | 7(1.4) | 543 | 12(2.2) | 0.61(0.24-1.56) | 0.31 |  | 0.73(0.28-1.91) | 0.52 |  | 0.90(0.34-2.40) | 0.83 |  |

Note: Adjusted HR/RR in model 2 was adjusted covariates comprising age, sex, baseline NIHSS score, baseline mRS, application of lipid-lowering agents before events, and antiplatelet therapy assignment (clopidogrel-aspirin and aspirin alone). Adjusted HR/RR in model 3 was adjusted covariates comprising age, sex, baseline NIHSS score, baseline mRS, application of lipid-lowering agents before events, antiplatelet therapy assignment (clopidogrel-aspirin and aspirin alone), hypertension, dyslipidemia, and previous ischemic stroke. The HRs are shown for stroke, composite cardiovascular events, ischemic stroke, moderate-to-severe bleeding, any bleeding, and all-cause death. The RRs are shown for poor functional outcome (mRS 2-6) and hepatotoxicity.

* Includes ischemic and hemorrhagic stroke.

# Includes stroke,myocardial infarction, or death from cardiovascular causes.

† Includes modified Rankin scale scores of 2 to 6 (range, 0 to 6, with higher scores indicating more disability and a score of 6 indicating death); data at 90 days were missing in 5 patients in the group of patients without diabetes mellitus, 1 patients in the group of patients with newly diagnosed diabetes mellitus, and 1 patients in the group of patients with a history of diabetes mellitus.

Abbreviations: HR, hazard ratio; RR, relative risk; T2DM, type 2 diabetes mellitus.
